# Apigenin Attenuates Paroxetine-Induced Ovarian Alterations in Female Rats

**DOI:** 10.3390/biology15100740

**Published:** 2026-05-07

**Authors:** Nazife Ulker Ertugrul, Tugrul Ertugrul, Feyza Keskin Buyukbudak, Ebru Gokdere, Meryem Sedef Dogru, Serife Tutuncu, Sinan Canpolat

**Affiliations:** 1 Department of Physiology, Faculty of Medicine, Samsun University, 55080 Samsun, Turkey; 2 Department of Histology and Embryology, Faculty of Veterinary Medicine, Ondokuz Mayıs University, 55200 Samsun, Turkey; tugrul.ertugrul@omu.edu.tr (T.E.); feyza.keskin@omu.edu.tr (F.K.B.); serife.tutuncu@omu.edu.tr (S.T.); 3 Experimental Research Center, Firat University, 23119 Elazig, Turkey; egokdere@firat.edu.tr; 4 Department of Physiology, Faculty of Medicine, Firat University, 23119 Elazig, Turkey; ms.dogru@firat.edu.tr; 5 Department of Physiology, Faculty of Medicine, Dokuz Eylul University, 35220 Izmir, Turkey; sinan.canpolat@deu.edu.tr

**Keywords:** paroxetine, apigenin, female reproductive toxicity, ovary, AMH, iNOS, VEGF, rat

## Abstract

Paroxetine, a selective serotonin reuptake inhibitor commonly used to treat various psychiatric disorders, may adversely affect female reproductive function. Although apigenin has been shown to ameliorate reproductive abnormalities and ovarian dysfunction, its effect on paroxetine-induced reproductive toxicity in females remains unclear. Therefore, this study investigated the potential protective effects of apigenin against paroxetine-induced reproductive alterations in female rats. Female rats with regular estrous cycles were randomly assigned to four groups (*n* = 9 per group): control, apigenin, paroxetine, and paroxetine + apigenin. The rats received saline, apigenin (20 mg/kg), paroxetine (10 mg/kg), or their combination by oral gavage once daily for about 29 consecutive days. Compared with paroxetine treatment alone, apigenin co-administration restored decreased serum anti-Müllerian hormone (AMH) levels, enhanced PAS reactivity in the zona pellucida, reduced ovarian iNOS immunoreactivity, increased follicle and corpus luteum numbers, and increased ovarian VEGF immunoreactivity. However, apigenin administration alone was associated with reduced testosterone levels and alterations in certain ovarian and uterine histological features in female rats. In conclusion, the findings suggest that apigenin may ameliorate paroxetine-induced reproductive alterations in female rats by modulating AMH levels, follicle and corpus luteum numbers, and ovarian histochemical and molecular parameters.

## Simple Summary

Paroxetine is a widely prescribed psychotropic medication used to treat depression and other psychiatric disorders, as well as some non-psychiatric conditions, but it may negatively affect female reproductive health. In this study, we investigated whether apigenin, a natural compound found in fruits, vegetables, herbs, and plant-based beverages, could mitigate these effects in female rats. Paroxetine treatment was associated with changes which suggested impaired ovarian function, including decreased serum anti-Müllerian hormone levels associated with ovarian reserve, reduced numbers of developing ovarian follicles, reduced numbers of corpora lutea, and structural alterations in ovarian tissue. When apigenin was co-administered with paroxetine, many of these changes were attenuated, suggesting that apigenin may partially exert a protective effect on ovarian function. At the same time, apigenin administration alone was associated with certain adverse effects, including reduced serum testosterone levels and histological alterations in reproductive tissues. These findings suggest that apigenin may help attenuate certain reproductive alterations associated with paroxetine, particularly by modulating paroxetine-induced ovarian changes. However, its use alone should be carefully considered. This work may help guide future studies on strategies to mitigate antidepressant-induced reproductive effects.

## 1. Introduction

Selective serotonin reuptake inhibitors (SSRIs) are commonly prescribed psychotropic medications for treating various psychiatric and non-psychiatric conditions due to their proven efficacy and favorable safety profile [1]. The use of SSRIs can lead to various side effects, including appetite disturbances, nausea, sleep disturbances, headaches, and reproductive and sexual adverse effects. Sexual side effects are one of the main reasons for early discontinuation of the medication [2,3,4]. Among the SSRIs, paroxetine is the most potent serotonin reuptake inhibitor and is the one most likely to cause sexual side effects such as decreased libido and vaginal lubrication difficulties in women. It has also been reported that paroxetine may have adverse effects on the female reproductive system, particularly by affecting fertility and inducing abnormal uterine contractions, precocious puberty, and premenstrual syndrome [5,6,7,8].

As SSRIs are prescribed long-term for depression or other mood and anxiety disorders, addressing treatment strategies for their adverse effects on the reproductive system, including potential sexual dysfunction, has become increasingly important. Strategies to manage SSRI-induced sexual dysfunction include pharmacological options such as dose reduction and switching antidepressants, as well as behavioral methods like exercise and psychotherapy, and complementary and integrative approaches such as nutraceuticals and acupuncture [7,9]. In complementary and integrative treatments, the use of maca root, saffron, and Rosa damascena oil as herbal agents has been shown to improve sexual function in women taking antidepressants [10,11,12]. In addition to addressing sexual dysfunction, studies indicate that maca root also has potential benefits for female reproductive health. Furthermore, saffron has been shown to alleviate symptoms associated with reproductive disorders in women [13,14].

In recent years, growing concerns about various health problems, including reproductive disorders and hormone-related diseases, have led to increased interest in herbal dietary supplements to prevent these diseases and alleviate their associated symptoms [15,16]. Apigenin is among the most extensively studied flavonoids for human health and is a phytoestrogen present in different plants at varying concentrations. Apigenin, chemically known as 4′,5,7-trihydroxyflavone, is abundant in medicinal plants, functional foods, fruits, and vegetables [17,18]. Numerous studies indicate that apigenin exhibits anti-cancer, anti-inflammatory, anti-mutagenic, antiviral, and laxative properties, and also contributes to free radical scavenging activity through its antioxidant properties [19]. Research suggests that apigenin may also impact female reproductive processes and associated abnormalities, although the available data are heterogeneous. For example, research indicates that apigenin can increase ovarian follicle number by modulating oxidative stress and apoptosis in ovarian cells [20]. Apigenin has also been reported to be involved in hormone regulation and the enhancement of ovarian function [21]. In contrast, a study in rats indicates that apigenin exhibits dose-dependent anti-implantation activity, and both flavonoids, apigenin and luteolin, are antifertility factors present in *S. orobanchioides* extract [22]. Nevertheless, it has also been reported that apigenin may have a mitigating or therapeutic effect on various reproductive system disorders such as polycystic ovary syndrome (PCOS) and ovarian dysfunction [18,20,23]. Despite the scientific evidence regarding the use of various herbal agents for treating reproductive disorders in women, the effect of the natural bioactive flavonoid apigenin on mitigating the adverse effects of SSRI antidepressants on female reproductive health remains unclear.

Recently, various physiological and pathophysiological processes, some of which are related to reproduction, have been linked to nitric oxide (NO). NO, synthesized by ovarian cells in both rats and humans, has become a crucial autocrine/paracrine regulator of ovarian physiology, including folliculogenesis, ovulation, steroidogenesis, corpus luteum function, implantation, and apoptosis. NO is produced from arginine by nitric oxide synthase (NOS). Inducible NOS (iNOS), which is also present in rat ovaries, is a significant isoform of this enzyme. In particular, iNOS is primarily expressed and localized in primary, secondary, and small antral follicles, as well as luteal cells [24,25]. NO has been shown to exert an inhibitory effect on androstenedione secretion and to have a pro-apoptotic effect in the human corpus luteum [24,26]. In addition, research has indicated that a significant increase in ovarian iNOS expression can be cytotoxic and may contribute to ovarian injury. However, a decrease in ovarian iNOS expression has been shown to be important in attenuating experimentally induced ovarian tissue damage in rats [27,28].

Angiogenesis is a critical process in female reproductive organs, and vascular endothelial growth factor (VEGF), a potent pro-angiogenic factor, plays a crucial role in the normal ovarian cycle. VEGF mRNA and protein are found in the uterus, ovary, and pituitary, which are components of the hypothalamic–pituitary–gonadal axis. VEGF contributes to the development of the corpus luteum and corpus albicans and increases vascular permeability in the thecal layer prior to ovulation [29,30]. Therefore, suppression of VEGF expression negatively affects follicle growth and ovulation, while increased VEGF expression may contribute to the restoration of ovarian function [31,32].

To fill a gap in the literature, our study aimed to elucidate the effects of apigenin, a common dietary flavonoid, on SSRI-induced reproductive toxicity in female rats. Furthermore, given the heterogeneity in the effects of apigenin on the female reproductive system, this study also sought to clarify its effects on the reproductive system in female rats. Accordingly, we hypothesized that chronic paroxetine exposure impairs ovarian reserve and folliculogenesis in regularly cycling female rats through the dysregulation of oxidative-inflammatory signaling and ovarian angiogenic balance, as reflected by altered iNOS and VEGF expression, reduced AMH, and histomorphological damage. We further hypothesized that apigenin co-administration would mitigate these alterations by suppressing iNOS-associated nitrosative stress, preserving VEGF-mediated follicular vascular support, and restoring ovarian reserve markers and follicular architecture.

## 2. Materials and Methods

### 2.1. Experimental and Stimulus Animals

Thirty-six experimental female Sprague–Dawley rats (2–3 months old, 200–300 g) showing two consecutive regular estrous cycles of 4–5 days were housed in the Firat University Experimental Animals Unit, Elazig, Turkey, at a temperature of 21 ± 1 °C and relative humidity of 50–55%, with a 12 h light/12 h dark cycle (lighting from 07:00 to 19:00). The animals were housed in groups of three in polycarbonate cages with unrestricted access to food and water. The experiment was approved by the Animal Experimental Ethics Committee of Firat University (No. 2024/11-05) and carried out in compliance with Firat University’s regulations for the care and use of laboratory animals.

In behavioral tests, an additional two male Sprague–Dawley rats (sexually experienced, gonado-intact, and 3–4 months old) were used as sexual stimuli. Four stimulus female Sprague–Dawley rats (ovariectomized, sexually receptive, and 2–3 months old) were used as social stimuli. These stimulus females underwent ovariectomy to eliminate hormonal fluctuations and were hormonally primed to ensure consistent sexual receptivity during behavioral testing. Specifically, ovariectomized female stimuli were made sexually receptive through subcutaneous injections of 10 μg estradiol benzoate and 500 μg progesterone, administered 48 h and 4 h before the behavioral tests, respectively. Estradiol benzoate and progesterone were dissolved in 0.1 mL sesame oil [33]. These stimulus animals were also obtained from the Firat University Experimental Animals Unit.

### 2.2. Experimental Groups

To test the hypothesis that apigenin protects against paroxetine-induced ovarian dysfunction, a controlled randomized parallel-group experimental design was employed. Female rats with verified regular estrous cyclicity were allocated into four treatment groups to differentiate: (i) basal ovarian physiology, (ii) the independent reproductive effects of apigenin, (iii) paroxetine-induced ovarian toxicity, and (iv) the potential protective effects of apigenin during paroxetine exposure. For this purpose, the experimental rats were randomly assigned as follows (*n* = 9 per group): (1) the control group, receiving 0.9% saline solution (1 mL/kg) orally; (2) the apigenin group, receiving 20 mg/kg apigenin orally; (3) the paroxetine group, receiving 10 mg/kg paroxetine orally; (4) the paroxetine + apigenin group, receiving both 10 mg/kg paroxetine and 20 mg/kg apigenin orally. The sample size per group was calculated using power analysis (power = 0.88, α = 0.05, effect size = 0.65) performed with G*Power software version 3.1.9. Saline, paroxetine, and/or apigenin were administered daily between 13:00 and 14:00 for an average of 29 days, until the rats were sacrificed. In the present study, the dosage and duration of apigenin and paroxetine were selected based on previously published protocols [34,35]. Paroxetine and apigenin were freshly prepared daily for each rat as saline suspensions (1 mL/kg) and thoroughly mixed to obtain uniform suspensions before oral administration. Body weights of rats in each experimental group were recorded daily during the entire experiment.

### 2.3. Estrous Cycle Monitoring

Vaginal secretions were collected daily from the rats for 10 days, starting on the seventh day of the experiment (days 7–16). Since evaluation of estrous cycle stages over several consecutive days enables short- and relatively longer-term monitoring of reproductive cyclicity in female laboratory rodents [36], the estrous cycle was monitored for 10 days, covering approximately 2–3 cycles. To examine the vaginal epithelium’s cytological morphology under a light microscope (Nikon YS100, Nikon, Tokyo, Japan), vaginal smears were collected between 14:00 and 15:00, as previously described. The rat estrous cycle consists of four stages: diestrus 1 (also referred to as metestrus), diestrus 2, proestrus, and estrus, and the complete cycle typically lasts 4–5 days. The stages of the estrous cycle were determined based on the predominant cell types observed in vaginal smears, including leukocytes, nucleated epithelial cells, and cornified cells, and their relative proportions [37].

### 2.4. Behavioral Tests

Tests for sexual incentive motivation and active investigation were performed between the 20th and 28th days of treatment, depending on the estrus phase of the rats. To ensure cycle consistency, vaginal cytology samples were collected immediately prior to each behavioral test to verify the estrus phase on the day of testing. The details of the testing arena and procedure were described previously in our study [38]. Briefly, the three-compartment testing apparatus consisted of a rectangular arena (91.44 × 31.75 cm) with walls 40 cm high. The female experimental rat was placed in the central compartment of the apparatus, while sexual and social stimuli were positioned in separate right and left compartments, each measuring 16.51 cm in length. Although physical contact between the female experimental rat and stimulus animals was not possible, the animals could see, hear, and smell each other through the Plexiglas barriers containing 1-cm-diameter holes between the compartments. The sexual incentive motivation test and active investigation test were conducted concurrently in the testing apparatus in all experimental female rats in the estrus phase. Each test was video-tracked by a night vision camera system for 10 min during the inverse dark phase of the light–dark cycle (between 15:00 and 17:00, in dim red light).

Before the behavioral tests, all female experimental rats were individually acclimated to the testing apparatus for two days (30 min per day), without sexual or social stimuli in the apparatus. Similarly, they were given an additional 5-min acclimation period right before the test on the test day. After each test, the apparatus was thoroughly cleaned using 70% ethanol to eliminate any residual urine, feces, or odors.

#### 2.4.1. Sexual Incentive Motivation Test

On the day of the test, immediately after a five-minute adaptation period, the intact female experimental rat was placed in its own cage. The sexual and social stimuli were then put individually in the other side compartments. Next, the female experimental rat was returned to the main compartment of the apparatus and permitted to move freely for 10 min. In a 10-min sexual incentive motivation test, the female experimental rat was observed spending time near the sexual or social stimulus when all four of its paws were within a 12.7 cm zone next to the barrier separating it from that stimulus. In this test, time spent near the male rat (TWM), time spent near the female rat (TWF), total social time (TST = TWM + TWF), and male preference ratio (TWM/TST) were computed.

#### 2.4.2. Active Investigation Test

During a 10-min active investigation test, the intact female experimental rat’s active exploratory behaviors in response to sexual or social stimuli were noted. These behaviors involve licking, sniffing, chewing, and climbing on barriers to reach sexual or social stimuli. Accordingly, duration of investigating the male rat (TIM), duration of investigating the female rat (TIF), total investigation time (TIT = TIM + TIF), and male investigation preference ratio (TIM/TIT) were computed. Using a stopwatch, the collected videos were manually scored by a researcher blinded to the experimental conditions. After completing the behavioral tests, the intact female experimental rats were returned to their home cages.

### 2.5. Sample Collection

On the day following behavioral testing, vaginal cytology was performed on all intact female experimental rats. To minimize hormonal variability associated with cycle stage, all terminal sampling was standardized to the diestrus–metestrus transition window, verified by vaginal cytology on the morning of sacrifice, and the animals were then sacrificed by direct decapitation (between days 21 and 29 of the experiment). Diestrus and metestrus were considered together during analysis, as these stages are reported to be similar in both cytology and hormone profiles [39]. Blood samples were collected from the trunk of the animals approximately 30–60 min after the last saline, paroxetine, and/or apigenin administration. Blood samples were then centrifuged at 4 °C at 4500 rpm for 5 min, and the resulting serum was aliquoted and stored at −80 °C until hormone analyses were performed. Furthermore, for histological and immunohistochemical analyses, the ovarian and uterine tissues were cleaned of excess fat and then fixed in a 10% formaldehyde solution.

### 2.6. Detection of Sex Hormones by ELISA

Serum levels of 17β-estradiol (E2) (201-11-0177; SunRed, Shanghai, China), progesterone (201-11-0742; SunRed, China), testosterone (201-11-5126; SunRed, China), prolactin (E-EL-R3006; Elabscience, Houston, TX, USA), and anti-Müllerian hormone (AMH) (E-EL-R3022; Elabscience, USA) were quantified using validated rat-specific enzyme-linked immunosorbent assay (ELISA) kits, following the manufacturer’s guidelines, and analyzed with an ELISA microplate reader (Multiskan FC, Thermo Scientific, Waltham, MA, USA). The assay sensitivities for E2, progesterone, testosterone, prolactin, and AMH were 0.752 ng/L, 1.115 ng/mL, 8.775 pg/mL, 1.88 ng/mL, and 37.5 pg/mL, respectively.

### 2.7. Histomorphological Analysis of the Ovary and Uterus

The ovaries and the medial portion of the uterine horns were dehydrated and then embedded in paraffin blocks using standard histological procedures. Five serial cross-sections, each 5 µm thick, were prepared from each paraffin block at intervals of 30 µm to avoid repeated evaluation of the same ovarian and uterine histological structures in consecutive sections. After mounting ovarian and uterine sections, the ovarian sections were stained using Crossman’s triple staining and periodic acid Schiff (PAS) staining, while the uterine sections were stained using only Crossman’s triple staining. PAS staining was performed exclusively on ovarian sections to assess the intensity of the PAS reaction in the zona pellucida. This parameter was integrated with the other ovarian findings, including follicular reserve, corpus luteum number, and iNOS and VEGF immunoexpression, to provide a comprehensive ovarian evaluation. In contrast, uterine tissue was evaluated by general histological examination and histomorphometric measurements. Slides were assessed at 10× magnification and photographed with an examination microscope (Nikon Eclipse 50i, Nikon, Tokyo, Japan). For each animal, histological evaluations were performed on these sections, and the mean value for each variable was calculated. Histological counting of ovarian follicles (primordial, primary, secondary, and Graafian) and corpus luteum was performed in all ovarian sections. Ovarian follicles were categorized according to the morphological criteria established by Mazaud et al. [40]. In the ovarian sections, PAS reactions in the zona pellucida were evaluated as none (−), weak (+), moderate (++), and severe (+++). The thickness of the uterine epithelium and the uterine wall (including the endometrium, myometrium, and perimetrium) was measured. Histological and immunohistochemical analyses were independently performed by two blinded investigators.

### 2.8. Immunohistochemistry Staining of iNOS and VEGF in the Ovary

iNOS and VEGF expression and localization in the ovary were determined using immunohistochemistry, as described previously [41]. Concisely, immunohistochemical evaluations were conducted on five serial ovarian sections for iNOS and five separate sections for VEGF, taken at 30 µm intervals to avoid repeated evaluation of the same immunopositive cells; the mean value was then calculated for each animal. After paraffin removal, antigen retrieval was carried out on 5-µm sections of ovarian tissue. This was followed by an overnight incubation at 4 °C with rabbit polyclonal iNOS (1:100 dilution, Abcam, Cambridge, UK, ab3523) or mouse monoclonal VEGF (1:500 dilution, Santa Cruz Biotechnology, Dallas, TX, USA, sc7269) primary antibodies. A goat anti-polyvalent horseradish peroxidase-conjugated antibody (Patolab, Istanbul, Turkey, PL-125-HL) and 3,3-diaminobenzidine chromogen (Patolab, PL-125-HD) were utilized to visualize immunoreactivity, following the manufacturer’s instructions. The sections were counterstained with hematoxylin, sealed with entallan, and then examined under a microscope.

Immunohistochemical scoring (histoscore) involved evaluating both the intensity and percentage of iNOS or VEGF immunostaining in the ovary under 20× magnification. The intensity of immunostaining was rated on a scale from 0 to 3: negative (0), trace (0.5), light (1), moderate (2), and intense (3). The percentage of iNOS- or VEGF-immunopositive area was scored on a scale of 0 to 0.9: 0 indicating no staining, 0.1 for less than 25% staining, 0.4 for 26–50% staining, 0.6 for 51–75% staining, and 0.9 for 76–100% staining, which is near homogeneity. To evaluate both the intensity and uniformity of iNOS or VEGF immunostaining at the same time, a composite histoscore was calculated. This histoscore is determined by multiplying the average intensity value for each tissue by the average percentage of stained area in each tissue (histoscore = intensity × area) [42].

The primary outcome measures were serum AMH concentration and total ovarian follicle count, as these directly reflect ovarian reserve and folliculogenesis. Secondary outcomes included iNOS and VEGF immunoreactivity, corpus luteum number, PAS reactivity, uterine morphometry, and behavioral indices. Together, the selected outcome measures were chosen to provide a multidimensional assessment of female reproductive integrity. This integrative framework enabled mechanistic interpretation of ovarian toxicity beyond isolated histological observations.

### 2.9. Statistical Analysis

The normality of data distribution was assessed using the Shapiro–Wilk test, and homogeneity of variances was evaluated with Levene’s test. All datasets, including body weight, estrous cycle patterns, behavioral test results, serum sex hormone levels, histological measurements, and immunohistochemical scores, were initially evaluated for normality and homogeneity of variances. For data that were normally distributed and showed equal variances, one-way analysis of variance (ANOVA) was applied, followed by Tukey’s post hoc test. For data that were normally distributed but did not show homogeneity of variances, one-way ANOVA was followed by Tamhane’s T2 post hoc test. When the assumption of normality was not met, the Kruskal–Wallis H test was used. Thus, the appropriate statistical test was applied for each analysis, taking into account the specific characteristics of the relevant dataset. Data were expressed as mean ± SEM (standard error of the mean). The correlation between serum AMH levels and total follicle number was assessed using Spearman’s correlation coefficient. Statistical analyses were performed using SPSS version 15.0. For all statistical tests, a *p*-value < 0.05 was considered statistically significant.

## 3. Results

### 3.1. Body Weight and Estrous Cycle

As expected, there was no significant difference in body weight between the control and experimental groups before administration of paroxetine and/or apigenin. Likewise, after 20 days of paroxetine and/or apigenin administration, the body weight of female rats treated with paroxetine and/or apigenin was also found to be similar to that of saline-treated female rats (Table 1).

Table 1 indicates no significant change in the frequencies of metestrus-diestrus, proestrus, and estrus phases in rats administered paroxetine and/or apigenin compared to rats in the control group.

### 3.2. Sexual Incentive Motivation and Active Investigation

To study the effects of the paroxetine and/or apigenin on sexual motivation, we compared parameters related to sexual incentive motivation and active investigation tests in the control and experimental groups. In the sexual incentive motivation test, no significant changes were observed in TWM, TWF, total social time, or male preference ratio in rats administered paroxetine and/or apigenin compared with the control group. Similarly, female rats’ active investigation behavior toward sexual or social stimuli was unaffected by paroxetine and/or apigenin administration compared to the control group, as evidenced by unchanged TIM, TIF, total investigation time, and male investigation preference ratio (Table 2). Thus, the sexual motivation of female rats was not affected by paroxetine and/or apigenin administration.

### 3.3. Serum Levels of Sex Hormones

**Figure 1:**
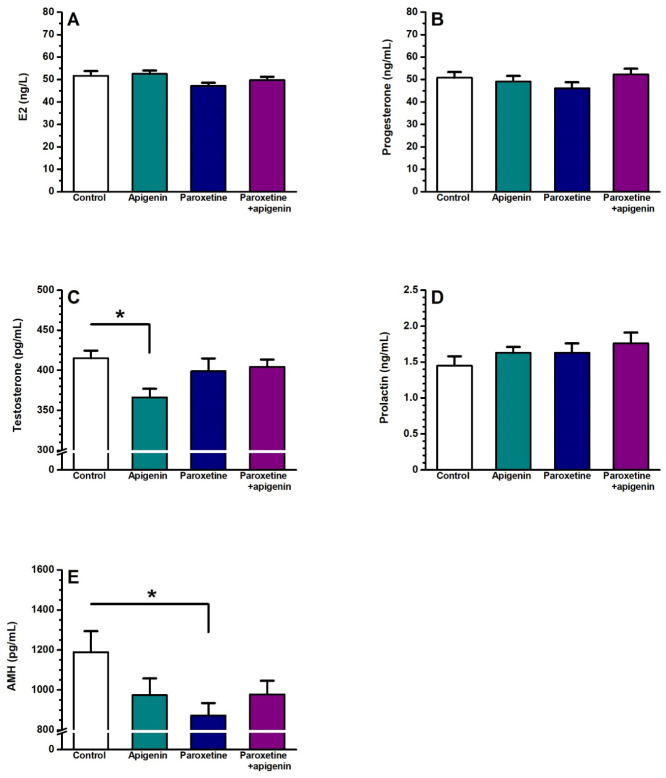
Effects of paroxetine and/or apigenin administration on serum levels of reproductive hormones in female rats. (**A**) 17β-estradiol (E2), (**B**) progesterone, (**C**) testosterone, (**D**) prolactin, (**E**) anti-Müllerian hormone (AMH). *n* = 9 rats per group. Data are presented as mean ± SEM, * *p* < 0.05 compared with the control group.

The effects of paroxetine and/or apigenin on serum sex hormones were assessed, and results revealed that paroxetine and/or apigenin administration in female rats had no significant effect on serum E2, progesterone, or prolactin levels compared with the saline-treated control group (Figure 1). On the other hand, only paroxetine administration led to a significant decrease in serum AMH levels (*p* < 0.05, Figure 1E), and only apigenin administration resulted in a statistically significant reduction in serum testosterone levels (*p* < 0.05, Figure 1C) when compared to the control group.

### 3.4. Ovarian Histomorphological Changes

**Figure 2:**
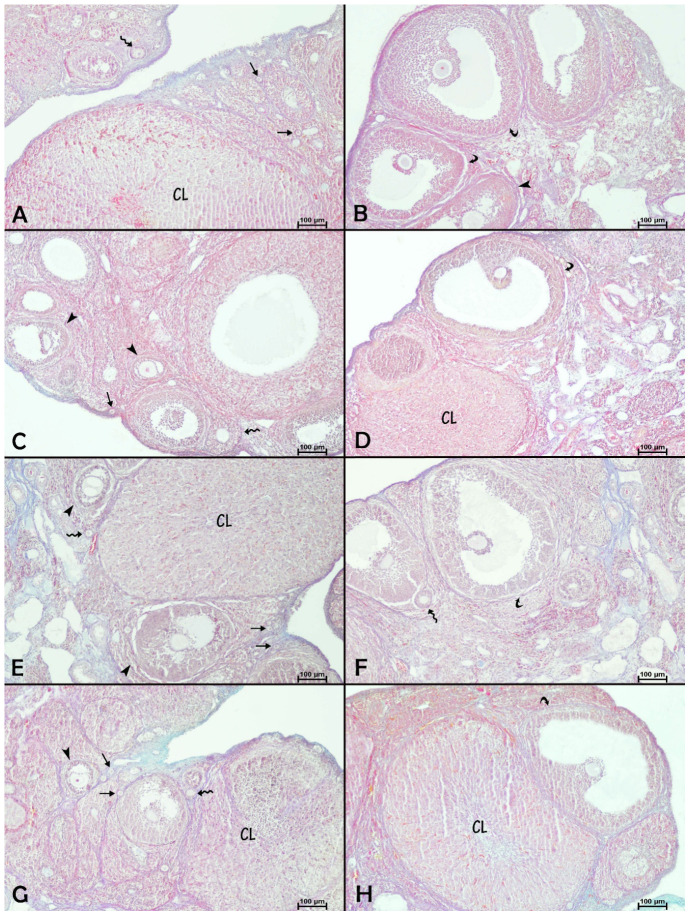
Effects of paroxetine and/or apigenin administration on ovarian histology in female rats. (**A**,**B**) Control group, (**C**,**D**) apigenin group, (**E**,**F**) paroxetine group, (**G**,**H**) paroxetine + apigenin group. Crossman’s triple staining, 10× magnification. Thin straight arrow, primordial follicle; zigzag arrow, primary follicle; arrowhead, secondary follicle; curved arrow, Graafian follicle; CL, corpus luteum.

Microscopic examination of Crossman’s triple-stained sections of the ovary from control and experimental groups was similar and showed the well-known standard structure of the ovary (Figure 2). Accordingly, it was noted that the ovaries were encased in a layer of germinal epithelium, and beneath this layer lies the tunica albuginea. The cortex was observed encircling the medulla beneath this layer. In the cortex, primordial, primary, secondary, and Graafian follicles were identified, and the granulosa cells surrounding the oocyte were clearly visible within these follicles. Furthermore, the corpus luteum, which was clearly visible, showed a standard histological structure (Figure 2).

Table 3 shows the histological counts of ovarian follicles and corpus luteum numbers in rats treated with saline and paroxetine and/or apigenin. The numbers of secondary and total follicles and the corpus luteum number were drastically decreased by apigenin administration compared with saline-treated rats (*p* = 0.001, *p* < 0.05, and *p* < 0.05, respectively). Compared with the control group, the numbers of primary and secondary follicles and corpus luteum in the paroxetine group were significantly lower (*p* < 0.05), along with a lower total follicle number (*p* < 0.01). In the paroxetine + apigenin group, apigenin administration significantly increased the number of primordial, primary, and total follicles (*p* < 0.05), as well as the corpus luteum number (*p* < 0.01), compared with the paroxetine group. These increases in corpus luteum number in the paroxetine + apigenin group were also statistically significant compared with the apigenin group (*p* < 0.01). In addition, Spearman’s correlation analysis showed no significant correlation between serum AMH levels and total follicle number (ρ = 0.092, *p* = 0.595).

**Figure 3:**
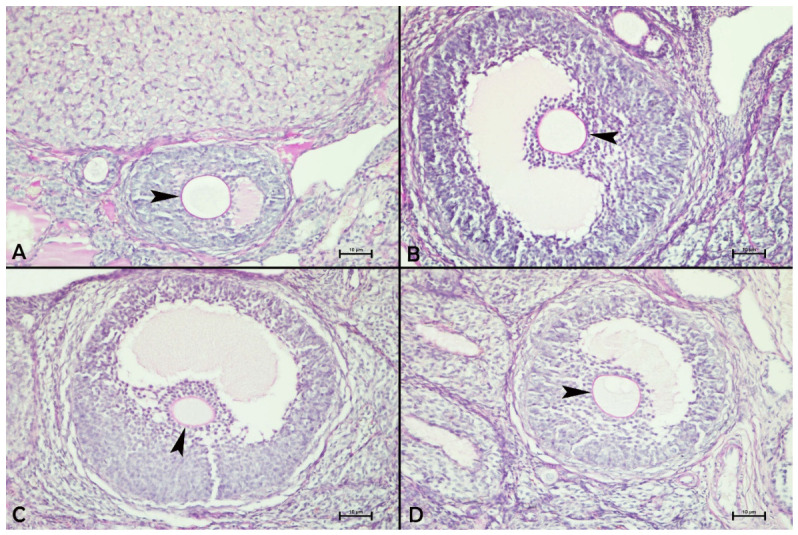
Effects of paroxetine and/or apigenin administration on ovarian PAS reactivity in the zona pellucida in female rats. (**A**) Control group, (**B**) apigenin group, (**C**) paroxetine group, (**D**) paroxetine + apigenin group. Periodic acid Schiff staining, 20× magnification. Arrowheads indicate the zona pellucida.

An examination of PAS-stained ovarian sections from the control, apigenin, and paroxetine + apigenin groups revealed that the zona pellucida exhibited intensely positive PAS reactions. In contrast, the zona pellucida of ovarian follicles showed faint PAS reactions in the paroxetine group compared to the other groups (Figure 3 and Table 4).

### 3.5. Uterine Histomorphological Changes

**Figure 4:**
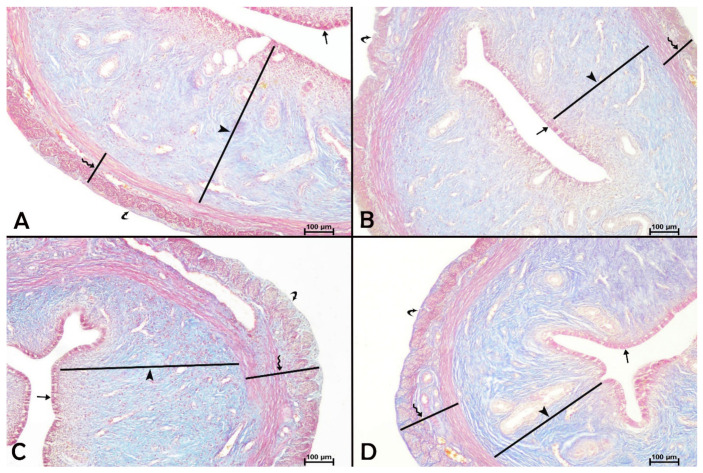
Effects of paroxetine and/or apigenin administration on uterine histology in female rats. (**A**) Control group, (**B**) apigenin group, (**C**) paroxetine group, (**D**) paroxetine + apigenin group. Crossman’s triple staining, 10× magnification. Straight arrow, epithelium; arrowhead, endometrium; zigzag arrow, myometrium; curved arrow, perimetrium.

We compared the rat uterine morphology in the control and experimental groups to identify any changes. Crossman’s triple staining results showed that the rats in the control, apigenin, paroxetine, and paroxetine + apigenin groups had normal uterine histology with the connective tissue fibers, uterine epithelium, and endometrium, including the uterine glands. Moreover, the myometrium, composed of smooth muscle cells arranged in a circular pattern on the inside and a longitudinal pattern on the outside, and the perimetrium, which surrounds this layer, had a standard histological structure (Figure 4).

The effects of paroxetine and/or apigenin on uterine morphometric measures are shown in Table 3. Results showed that paroxetine treatment or co-treatment with paroxetine and apigenin did not affect the thickness of the uterine epithelium or the uterine wall when compared to the saline-treated control group. However, the thicknesses of the uterine epithelium (*p* < 0.05), myometrium (*p* < 0.05), and perimetrium (*p* < 0.001) were significantly lower in the apigenin-treated rats than in the saline-treated controls. These reductions in the myometrial and perimetrial thickness in the apigenin group were also statistically significant compared to the paroxetine group (*p* < 0.01). Similarly, the thickness of the uterine epithelium and perimetrium of the apigenin-treated rats was significantly lower than that of rats co-administered with paroxetine and apigenin (*p* < 0.01 and *p* < 0.05, respectively).

### 3.6. Immunohistochemical Analysis of iNOS and VEGF Expression in Ovaries

**Figure 5:**
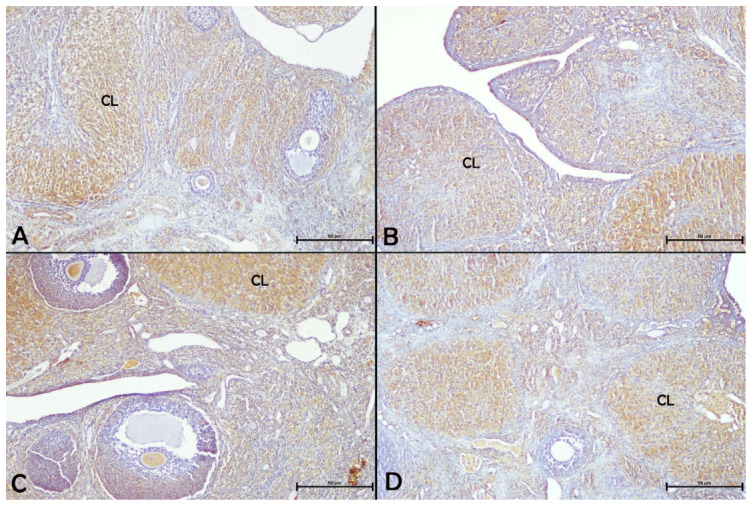
Effects of paroxetine and/or apigenin administration on ovarian iNOS immunoreactivity in female rats. (**A**) Control group, (**B**) apigenin group, (**C**) paroxetine group, (**D**) paroxetine + apigenin group. 10× magnification. CL: Corpus luteum.

Immunohistochemical analysis of iNOS expression in ovarian sections from all experimental groups demonstrated pronounced positive staining in granulosa cells of the follicles, interstitial cells, theca cells, luteal cells of the corpus luteum, and germinal epithelium (Figure 5). When ovarian iNOS immunoreactivity was evaluated across the experimental groups, no significant changes were observed in the apigenin or paroxetine + apigenin groups in comparison with the control group, whereas it was significantly increased in the paroxetine group compared with both the control and apigenin groups (*p* < 0.05, Table 4).

**Figure 6:**
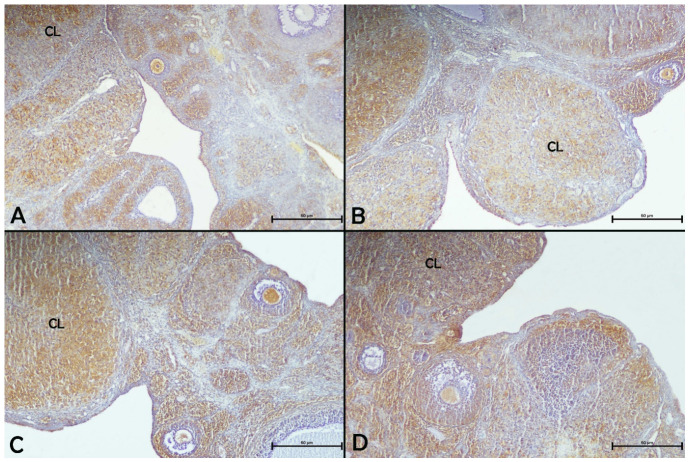
Effects of paroxetine and/or apigenin administration on ovarian VEGF immunoreactivity in female rats. (**A**) Control group, (**B**) apigenin group, (**C**) paroxetine group, (**D**) paroxetine + apigenin group. 10× magnification. CL: Corpus luteum.

VEGF immunopositivity was observed in granulosa cells of ovarian follicles, interstitial cells, luteal cells of the corpus luteum, and germinal epithelium across all experimental groups (Figure 6). The results showed that paroxetine and/or apigenin administration did not affect ovarian VEGF immunoreactivity compared with the control group. On the other hand, the co-administration of paroxetine and apigenin in the paroxetine + apigenin group led to a significant increase in ovarian VEGF immunoreactivity compared with the apigenin group (*p* < 0.05, Table 4).

## 4. Discussion

To date, no studies have investigated the effects of apigenin on paroxetine-induced alterations in the female reproductive system in rats. In this context, the present study is the first to explore the potential protective effects of apigenin against paroxetine-induced reproductive changes in female rats. According to our results, paroxetine and/or apigenin administration did not significantly affect body weight, estrous cycle, or sexual incentive motivation in female rats. However, apigenin alone decreased serum testosterone levels and was associated with alterations in certain ovarian and uterine parameters. Paroxetine treatment was associated with changes suggestive of impaired ovarian function, including decreased serum AMH levels, reduced follicle numbers, faint ovarian PAS reaction, and increased ovarian iNOS immunoreactivity. Notably, apigenin co-administration attenuated several of these paroxetine-induced alterations, with serum AMH levels and ovarian iNOS expression approaching control values, restoration of PAS reactivity in the zona pellucida, improvement in parameters related to folliculogenesis and luteal activity, and increased ovarian VEGF expression. Collectively, these results suggest that apigenin may exert partial modulatory effects against paroxetine-induced ovarian alterations in female rats, while also being associated with alterations in certain female reproductive parameters when administered alone.

SSRIs are among the most extensively prescribed antidepressant medications globally; however, their impact on the reproductive system is often overlooked [43]. Research indicates that long-term SSRI use may disrupt the estrous cycle, while other studies have reported that SSRIs have no effect on it. It has also been suggested that SSRI-related disruption of the estrous cycle may be associated with rat strain differences. For example, fluoxetine, another SSRI, was reported to disrupt the estrous cycle in Fischer rats, whereas it did not affect the estrous cycle in Wistar, Long–Evans, or Sprague–Dawley female rats [44]. In the present study, short-term administration of paroxetine had no effect on the estrous cycle in Sprague–Dawley rats. In this context, flavonoids are among the most prevalent and widely distributed polyphenolic compounds found in many plant-based foods, and evidence suggests that dietary flavonoid intake may impact the female reproductive system. Apigenin is a natural plant-derived flavonoid abundantly present in many common fruits and vegetables [18,45]. Apigenin, known for its phytoestrogenic properties, has shown potential protective effects on ovarian function [23]. For instance, apigenin has been shown to restore disrupted estrous cycles in experimental animal models caused by ovariectomy or PCOS [18,20]. However, there are limited studies reporting the effects of apigenin on the estrous cycle in normal cycling or paroxetine-treated rats. To our knowledge, the current study demonstrates for the first time that apigenin does not affect the estrous cycle in rats with regular estrous cycles or in those treated with paroxetine.

Kaspersen and Ågmo [35] demonstrated that 20-day administration of paroxetine (10 mg/kg) decreased sexual motivation in female rats. This study also revealed that when the duration of paroxetine treatment increased, the effect on sexual motivation diminished. Our study similarly found that approximately 29 days of paroxetine administration did not affect sexual motivation in female rats. In rodent models, flavonoids or extracts containing flavonoid derivatives have been observed to enhance sexual behavior, and free apigenin has been shown to have pro-sexual effects [46,47]. As far as we are aware, this study constitutes the first assessment of the effects of apigenin on sexual incentive motivation in female rats. Accordingly, our findings indicate that apigenin did not affect sexual motivation in either normal cycling or paroxetine-treated rats, and this outcome may depend on the dose or duration of apigenin administration.

In female rodents, sexual receptivity and motivation are regulated by cyclic changes in gonadal hormone release, particularly the coordinated sequential release of estradiol and progesterone [48]. In the present study, serum 17β-estradiol and progesterone levels did not change significantly following paroxetine and/or apigenin administration, which may help explain the lack of change in sexual motivation observed in the experimental groups.

AMH regulates follicular growth and development and is directly produced by granulosa cells of secondary, preantral, and early antral follicles in the ovary. There is a positive correlation between serum AMH levels and ovarian reserve (follicular pool) [49]. A study indicated that circulating AMH concentrations are positively correlated with the total number of healthy follicles and oocytes in the ovaries of mice. Furthermore, it has been reported that AMH concentrations decrease in parallel with the decline in follicle number in rodents [50]. Therefore, AMH is an endocrine marker that reliably indicates reproductive capacity. Recommended indicators for assessing reproductive function in rodents include serum AMH levels, as well as the estrous cycle and serum estradiol and follicle-stimulating hormone (FSH) levels [51,52]. Research indicates that serum AMH levels decrease in patients treated with SSRIs, and this decline has been linked to a lower baseline ovarian reserve or a higher rate of early pregnancy loss [53,54]. Similarly, in the present study, paroxetine administration significantly reduced serum AMH levels in female rats, suggesting a negative impact on ovarian reserve, an important indicator of female reproductive capacity. However, no significant correlation was found between serum AMH levels and total follicle number in the present study. This may be related to the treatment-specific pattern observed across experimental groups, in which serum AMH and total follicle number did not fully parallel each other across all groups.

It has been reported that reproductive processes (including ovarian functions) can be regulated by the integration of various factors, including plant flavonoids such as apigenin [55]. Apigenin has been shown to mitigate the effects of endocrine disruptors on reproductive health [56]. Peng et al. [20] reported that apigenin treatment improved the hormonal profile by preserving levels of reproductive hormones such as estrogen, testosterone, and progesterone in PCOS rats. In ovariectomized mice, oral apigenin treatment has also been shown to modulate serum estrogen levels [18]. In a recent study, apigenin has been shown to reverse decreases in serum AMH concentrations and increases in serum luteinizing hormone (LH) and FSH concentrations in a mouse model of perimenopausal syndrome [23]. Despite this, no studies have investigated the impact of apigenin on paroxetine-induced changes in sex hormone levels. In line with previous findings, the current study demonstrated that the paroxetine-induced reduction in serum AMH levels was attenuated by apigenin co-administration, resulting in AMH levels approaching those of the control group. These findings indicate that apigenin may modulate the endocrine-related effects of paroxetine treatment in female rats.

In the present study, the adverse effect of apigenin on the female reproductive endocrine system was evidenced by a decrease in serum testosterone levels in apigenin-treated female rats. Notably, inconsistent findings regarding the reproductive effects of apigenin have been reported in the literature. Many studies have shown the beneficial effects of apigenin on the reproductive system and related dysfunctions [18,20,21,23]. Moreover, research supporting our findings has reported that apigenin is a potent inhibitor of steroidogenic enzymes in both rats and humans [57]. In another study by Hiremath et al. [22], apigenin was shown to exhibit dose-dependent anti-implantation activity, potentially due to an imbalance in endogenous estrogen and progesterone levels. Additionally, apigenin has been reported to adversely affect the reproductive system due to its ability to reduce sperm density, inhibit spermatogonial proliferation, and induce spermatogonial apoptosis [58,59].

The mammalian zona pellucida is an important extracellular matrix consisting of a transparent glycoprotein layer surrounding the oocyte [60]. PAS staining is a histochemical method used to detect carbohydrate stores and the integrity of the zona pellucida and oocyte. Studies have shown that faint PAS reactions and reduced PAS optical density in the zona pellucida reflect a reduction or even depletion of carbohydrates in the oocytes and the surrounding zona pellucida [61]. Moreover, it has been suggested that abnormalities in the zona pellucida may contribute to infertility observed in female rats [62]. In the present study, faint PAS reactions were observed in the zona pellucida in PAS-stained ovarian sections of paroxetine-treated rats compared to the control group. However, in rats co-treated with paroxetine and apigenin, PAS reactivity in the zona pellucida was more intense than that observed in paroxetine-treated rats. In parallel with faint PAS reactions in the zona pellucida and decreased serum AMH levels, paroxetine-treated rats also showed decreased numbers of ovarian follicles (primary, secondary, and total) and corpora lutea. Taken together, these results suggest that paroxetine has an adverse effect on ovarian tissue. Consistent with our study, previous research has also shown that several SSRIs, particularly paroxetine, fluoxetine, or escitalopram, have adverse histological effects on rat ovarian tissue, evidenced by decreases in ovarian follicle numbers (primordial, primary, or secondary) or in corpora lutea [63,64].

Bhardwaj et al. [65] reported that a decrease in follicular count can lead to premature ovarian failure and female infertility. A decrease in the number of corpora lutea reflects an inhibitory effect on luteal function or ovulation [66]. In contrast, a high number of corpora lutea indicates a healthy ovulation process, correlating with an increase in ovulation rate [67]. Our study found that apigenin significantly decreased the number of secondary and total follicles and the number of corpora lutea in rats. All these data suggest a potentially detrimental role of apigenin in the regulation of ovarian function, including steroidogenesis, folliculogenesis, and ovulation. In addition to its effects on ovarian tissue, this study demonstrated that apigenin caused reductions in the thickness of the uterine epithelium, myometrium, and perimetrium. In the current study, while apigenin was observed to have adverse effects on the reproductive tract of normal cycling rats, it attenuated paroxetine-induced alterations in the ovaries, as evidenced by increased ovarian follicle and corpora lutea numbers, along with stronger PAS reactivity in the zona pellucida. Thus, our findings indicate that apigenin may improve folliculogenesis and modulate ovulation-related parameters in paroxetine-treated rats.

Because apigenin has been proposed as a safe and promising therapeutic agent for the treatment of ovarian dysfunction [18], ovarian VEGF expression was one of the main focuses of the present study. VEGF is known to play a crucial role in angiogenesis and to regulate vascular development within ovarian follicles. A positive correlation between follicular diameter and VEGF expression has also been reported. Suppression of VEGF expression disrupts follicle growth and ovulation, while a significant increase in VEGF expression levels promotes ovarian angiogenesis [31,68]. It has also been reported that high VEGF expression may play a crucial role in restoring ovarian function [32]. In the present study, we found that ovarian VEGF immunoreactivity was significantly higher in the paroxetine + apigenin group than in the apigenin group. In parallel with this increase, the number of corpora lutea was also higher in the paroxetine + apigenin group compared with the apigenin group. These data suggest that increased VEGF expression may contribute to the restorative effects of apigenin on paroxetine-induced ovarian alterations.

Increased iNOS expression suggests elevated NO levels and is associated with cytotoxic effects. Specifically, it has been reported that upregulation of ovarian or testicular iNOS expression may exert deleterious effects on reproductive tissues, thereby contributing to tissue injury [27,28,69,70]. Beltrame et al. [69] reported that strong immunostaining for iNOS was observed in testicular tissues of rats treated with paroxetine. Similarly, paroxetine treatment has been shown to increase iNOS levels in rat penile tissue, and this effect is prevented by tadalafil treatment [71]. To the best of our knowledge, the present study is the first to examine the effect of paroxetine on iNOS expression in rat ovaries. Accordingly, paroxetine administration significantly increased iNOS expression in rat ovarian tissue, which is consistent with findings reported in male reproductive models. In our study, increased iNOS expression in the ovaries of paroxetine-treated rats suggests that excessive NO production mediated by iNOS may contribute to ovarian tissue alterations associated with paroxetine exposure. On the other hand, downregulation of iNOS expression is known to exert protective effects against ovarian tissue damage [28]. Additionally, increased iNOS expression has been observed in the peritoneal fluid of women with infertility due to endometriosis. Moreover, therapeutic strategies targeting the reduction of iNOS expression have been suggested to be beneficial in preventing infertility in these women [72]. Perimenopause is a transitional phase in middle-aged women characterized by a gradual decline in ovarian function and irregular menstrual cycles. In this respect, recent studies have demonstrated a significant increase in ovarian iNOS expression in a mouse model of perimenopausal syndrome. Furthermore, this increase was significantly reversed by apigenin; therefore, it has been proposed that apigenin may ameliorate ovarian dysfunction [23]. Consistent with this study, we found that the paroxetine-induced upregulation of ovarian iNOS immunoreactivity was reversed by apigenin co-administration in the paroxetine + apigenin group, bringing it to levels comparable to those observed in the control group. In conclusion, our study suggests that the apigenin-induced downregulation of iNOS immunoreactivity in rat ovaries may contribute to the protection of ovarian tissue against paroxetine-induced damage.

In the present study, paroxetine and/or apigenin administration did not significantly affect body weight in female rats. Examination of paroxetine’s effects on body weight reveals inconsistent findings across experimental animal models, with some indicating an increase while others show no change. Notably, biological sex, dosage, and duration of treatment have been identified as factors that influence these outcomes [73,74,75]. Similarly, a review of relevant literature shows that apigenin’s effects on body weight are variable and appear to be dose- and time-dependent. Alamri [76] reported that an increase in body weight was observed in rats administered apigenin at a dose of 50 mg/kg for 3 weeks. Conversely, numerous studies indicate that different doses (i.e., 10, 25, or 50 mg/kg) and durations (i.e., 2, 8, or 12 weeks) of apigenin administration do not significantly affect body weight in rodent models [77,78,79]. Consistent with these findings, no significant change in body weight was observed in female rats treated with apigenin (20 mg/kg) for approximately 29 days in the present study. Additionally, apigenin treatment did not alter body weight in paroxetine-treated female rats. Overall, the findings of the present study suggest that the administration of paroxetine and apigenin may exert adverse effects on the reproductive system of female rats independently of body weight changes.

First, a limitation of the present study is that paroxetine and apigenin were evaluated at a single dose level; therefore, the dose-dependent and potentially differential effects of apigenin could not be determined. Second, because oxidative stress-related biomarkers were not measured, the possible contribution of apigenin’s antioxidant activity to the observed effects could not be evaluated, and the potential involvement of other relevant pathways, including apoptosis-related signaling pathways and estrogen receptor-mediated signaling, remains unclear. Third, stress-related biomarkers were not assessed; therefore, the potential contribution of repeated oral gavage-induced stress to the observed findings cannot be ruled out.

## 5. Conclusions

In conclusion, this study suggests that apigenin co-administration may improve selected reproductive parameters affected by paroxetine in female rats, with effects observed in parameters such as serum AMH levels, ovarian iNOS and VEGF immunoreactivities, PAS reactivity in the zona pellucida, folliculogenesis, and luteal activity. Additionally, apigenin administration alone in female rats reduced serum testosterone levels and was associated with changes in specific ovarian and uterine histological features. Collectively, these findings indicate a potential modulatory effect of apigenin on paroxetine-induced reproductive alterations in female rats, particularly in relation to serum AMH levels and ovarian histological, histochemical, and molecular parameters.

## Figures and Tables

**Table 1:** Effects of paroxetine and/or apigenin administration on body weight and estrous cycle pattern in female rats. *n* = 9 rats per group. Data are presented as mean ± SEM.

Parameters	Control	Apigenin	Paroxetine	Paroxetine + Apigenin
Body weight (g)				
Initial	258.4 ± 3.4	253.6 ± 2.6	262.4 ± 3.1	261.9 ± 3
Day 20	280.8 ± 5.2	270.1 ± 4.8	273.8 ± 2.3	277.5 ± 4.3
Phases of estrous cycle				
Diestrus/metestrus frequency (%)	64.4 ± 6	61.1 ± 7	56.7 ± 5	57.8 ± 4.9
Proestrus frequency (%)	13.3 ± 2.9	20 ± 4.1	23.3 ± 2.9	20 ± 2.9
Estrus frequency (%)	22.2 ± 3.6	18.9 ± 3.1	20 ± 3.7	22.2 ± 2.8

**Table 2:** Effects of paroxetine and/or apigenin administration on sexual incentive motivation and active investigation in female rats. *n* = 9 rats per group. Data are presented as mean ± SEM.

Parameters	Control	Apigenin	Paroxetine	Paroxetine + Apigenin
Sexual incentive motivation				
Time spent near the male rat (TWM)	290.4 ± 21.9	242.1 ± 21.8	287.6 ± 42.9	286.2 ± 37
Time spent near the female rat (TWF)	180.9 ± 21.3	225.7 ± 19.6	174.6 ± 32.5	178.9 ± 26.5
Total social time (TST = TWM + TWF)	471.3 ± 11.1	467.8 ± 12.4	462.1 ± 20.7	465.1 ± 24.1
Male preference ratio (TWM/TST)	0.62 ± 0.04	0.52 ± 0.04	0.61 ± 0.08	0.61 ± 0.06
Active investigation				
Duration of investigating the male rat (TIM)	137.6 ± 17.8	142.1 ± 23.9	153 ± 26.3	126 ± 20.7
Duration of investigating the female rat (TIF)	74.2 ± 10.5	87.7 ± 11.2	64.1 ± 12.2	54.3 ± 6.7
Total investigation time (TIT = TIM + TIF)	211.8 ± 19.5	229.8 ± 31.4	217.1 ± 21.8	180.3 ± 21.3
Male investigation preference ratio (TIM/TIT)	0.64 ± 0.05	0.6 ± 0.03	0.68 ± 0.07	0.68 ± 0.05

**Table 3:** Ovarian follicle and corpus luteum numbers, and uterine morphometric parameters in rats treated with saline, paroxetine and/or apigenin. *n* = 9 rats per group. Data are presented as mean ± SEM, * *p* < 0.05, ** *p* < 0.01, *** *p* = 0.001, and **** *p* < 0.001 compared with the control group; ^#^ *p* < 0.05 and ^##^ *p* < 0.01 compared with the apigenin group; ^$^ *p* < 0.05 and ^$$^ *p* < 0.01 compared with the paroxetine group.

Parameters	Control	Apigenin	Paroxetine	Paroxetine + Apigenin
Ovary				
Primordial follicle	7.1 ± 0.3	6.8 ± 0.4	6.5 ± 0.3	7.8 ± 0.2 ^$^
Primary follicle	1.3 ± 0.2	1.1 ± 0.1	0.74 ± 0.04 *	1.5 ± 0.2 ^$^
Secondary follicle	4.1 ± 0.2	3.1 ± 0.1 ***	3.4 ± 0.2 *	3.6 ± 0.2
Graafian follicle	0.78 ± 0.2	0.28 ± 0.1	0.58 ± 0.2	0.31 ± 0.1
Total follicle	13.3 ± 0.41	11.3 ± 0.5 *	11.2 ± 0.31 **	13.2 ± 0.51 ^$^
Corpus luteum	9.6 ± 0.5	7.7 ± 0.4 *	7.7 ± 0.3 *	10 ± 0.5 ^##, $$^
Uterus				
Uterine epithelium thickness (µm)	18.6 ± 0.5	16.3 ± 0.5 *	18.1 ± 0.3	19.6 ± 0.8 ^##^
Myometrial thickness (µm)	230.3 ± 12.4	188.7 ± 11.1 *	240.9 ± 10.8 ^##^	211.4 ± 6.5
Endometrial thickness (µm)	385.4 ± 19.4	388.7 ± 8.2	357.6 ± 8.2	361.6 ± 8.1
Perimetrium thickness (µm)	14.1 ± 0.7	10.8 ± 0.3 ****	13.1 ± 0.6 ^##^	12.8 ± 0.4 ^#^

**Table 4:** Ovarian PAS reaction and ovarian iNOS and VEGF immunohistochemical histoscores (area stained × intensity) in rats treated with saline, paroxetine, and/or apigenin. *n* = 9 rats per group. Data are presented as mean ± SEM, * *p* < 0.05 compared with the control group; ^#^ *p* < 0.05 compared with the apigenin group. (++): moderate; (+++): severe.

Parameters	Control	Apigenin	Paroxetine	Paroxetine + Apigenin
PAS reaction	+++	+++	++	+++
Histoscore of iNOS expression	0.67 ± 0.06	0.6 ± 0.12	1.42 ± 0.19 *^, #^	0.93 ± 0.14
Histoscore of VEGF expression	1.67 ± 0.09	1.2 ± 0.14	1.4 ± 0.16	1.8 ± 0 ^#^

## Data Availability

The original contributions presented in this study are included in the article. Further inquiries can be directed to the corresponding author.
